# 

**Published:** 1826-06

**Authors:** 


### MONTHLY CATALOGUE OF MEDICAL BOOKS.

*** *It having been hinted to us that gentlemen, sending copies of their Works, prefer having their titles given at length, it is our intention, in future, to comply with this suggestion: but it is to be observed, that no books can be entered on this List except those sent to us for the purpose;—finding, in the lists hitherto transmitted, that the names of works have frequently been given as published, which have not appeared for weeks, or even months, after.*

An Essay on Headachs, and on their Cure. By WALTER VAUGHAN, M.D. of the Royal College of Physicians in London.—London, 1825.

An Exposition of the State of the Medical Profession in the British Dominions; and of the injurious Effects of the Monopoly, by Usurpation, of the Royal College of Physicians in London.—London, 1826.

The Surgeon-Dentist's Anatomical and Physiological Manual. By G. WAITE, Member of the Royal College of Surgeons.—London, 1826.

Farther Remarks on Hernia, in Explanation of the Nature of Strangulation, and of obliterated Intestine, and in Defence of Views and Suggestions towards Improvement in the Treatment. By E. GEOGHEGAN, M.R.C.S. Honorary Member of the Royal Medical Society, Edinburgh; and Surgeon to the Dublin General Dispensary. In a Letter to JOHN ABERNETHY, Esq.—Dublin.

A Case of Melanosis, with general Observations on the Pathology of this interesting Disease. By THOMAS FAWDINGTON, Member of the Royal College of Surgeons, London; and one of the Surgeons to the Manchester Lying-in Hospital. Illustrated by coloured Lithographic Plates.—London, 1826.

Proceedings of the Seventh Anniversary Meeting of the Hunterian Society, held on the 8th of February, 1826 ; with the Report, and List of Officers and Members.—London, 1826.

### NOTICE TO CORRESPONDENTS.

*We request the attention of our Correspondents to the Prospectus at the commencement of this Number.—Communications, through the usual channel, are respectfully solicited.*

Bind this Line

London; May 30, 1826.

THE

### London Medical and Physical Journal,

EDITED BY DR. MACLEOD.

---

For many fortunate discoveries in medicine, and for the detection of numerous errors, the world is indebted to the rapid circulation of Monthly Journals; and there never existed any work, to which the Faculty, in Europe and America, were under deeper obligations, than to the Medical and Physical Journal of London, now forming a long, but an invaluable, series.—RUSH.

---

The Proprietors of this Journal beg to inform the Medical Profession, and the Public, that they intend to commence a New Series on the 1st of July. They have had this measure in contemplation for some time, in consequence of frequent applications for an opening, having been made to them by gentlemen desirous of becoming subscribers.

The circumstance, however, which has decided them in choosing the present period for carrying the measure into effect, is their desire to bring fully before the Profession certain improvements, which they trust will be regarded as essentially enhancing the value of their publication. These relate both to the matter and the manner in which it will be presented to the reader.

1st. An *entirely new Department* will be added, consisting of instructive CASES obtained from PUBLIC INSTITUTIONS, and other authentic sources. These, they have reason to believe, will constitute a mass of medical and surgical information, greatly exceeding in importance any thing of the kind which they have hitherto been able to procure.

2dly. The typography of the Journal will be much improved, as they have obtained NEW TYPES expressly for the purpose; while, in order to meet the additional quantity of matter, *eight pages* will be added to each Number; being an addition of *forty-eight pages* to the volume.

For the spirit in which the Work will be conducted, the Proprietors take leave to refer to the last few volumes. They have the best reasons for believing that the labours of the present Editors have met the approval of their professional brethren: reasons founded on the increase in the number and value of the Original Communications, as well as the extended sale of the Journal.

In other respects, no essential change will take place: the ORIGINAL PAPERS will be rendered still more select;—the CRITICAL ANALYSES of all important Works, both domestic and foreign, will be continued;—and the departments of COLLECTANEA and INTELLIGENCE will, as before, contain short notices of cases, and facts of every kind interesting to the Medical Profession, arranged according to subjects.

The *new Series* will constitute an immediate continuation of the *old*; the numbering of which will be continued, as well as that of the Series about to be commenced. The Journal will thus form a complete record of Medical Literature from the beginning of the present century.

Orders for the New Series will be received by all Booksellers; and those who intend to become Subscribers are respectfully requested to intimate their intentions as soon as possible, in order that the additional number of copies struck off may be made as nearly as possible to correspond with the demand.

The Proprietors beg to add, that, notwithstanding the very considerable expense incurred by these new arrangements, the price of the Journal will continue the same as before, viz. 2s. 6d. per Number.

ADVERTISEMENTS will be inserted on the Cover, and BILLS stitched in the Work, as hitherto, at the usual charges.

---

Published by J. SOUTER, 73, St. Paul's Church Yard; and may be had through the medium of all Booksellers, in Town and Country.

*J. and C. Adlard, Printers, Bartholomew Close.*

### INDEX

OF

THE FIFTY-FIFTH VOLUME

OF THE

### London Medical and Physical Journal.

PAGE

ABDOMINAL Pregnancy, case of 178
———— Dropsy, case of . 274
Abscess of the Œsophagus . 518
Account of Scurvy in Russia . 465
———— Diseases of the Eye . 378
Action of Calomel, on the . 40
Acupuncturation, marvellous effects of . . . . 174
Affection of the Brain, Schirrous, on 369
Alveolar Arches, extirpation of the 355
Analysis of Opium . . 179
———— Rhubarb . . 266
Anchylosis of the Knee-joint, case of 112
Aneurism, Popliteal, case of 271, 359
Animal Magnetism . . 429
Aorta, Aneurism of, case of . 430
Apoplexy, case of . . 190
Apparatus, a new Cupping . 356
Application of Lunar Caustic, efficacy of . . . . 364
Arsenic, detection of, in persons poisoned . . . 525
———— and Sulphate of Quina in Acute Rheumatism . . 106
Atmosphere, on Organic Exhalations in the . . . 442

Bandage, case of Dyspnœa, cured by . . . . 173
Bicarbonate of Soda as a Lithontriptic . . . . 522
Bile, experiments on the Secretion of . . . . 354
Bleeding in Pulmonary Inflammation . . . . 433
Blood-letting, on the utility of . 517
Bones, metallic Mercury in the 432
Brain, Schirrous Affection of the 369

Calomel, on the action of . . 40
———— a large dose given to a Child . . . . 433
Calculi, Salivary, observations on 519
Calculus in the Urethra . . 70
Case of Lithotomy, attended with Hemorrhage . . . 3

PAGE

Case of Headache . . 17
———— Small-Pox after Small-Pox Inoculation . . . 28
———— Epileptic Convulsions . 103
———— Poisoning by Opium . 109
———— Anchylosis of the Knee-joint . . . . 112
———— Tic Douloureux . . 123
———— Abdominal Pregnancy . 178
———— Apoplexy . . 190
———— Dropsy . . . 202
———— Enlargement of the Mammæ . . . . 260
———— Popliteal Aneurism 271, 359
———— Uterine Hemorrhage . 458
———— Abdominal Dropsy . 272
———— Diabetes . . . 366
———— Neuralgia . . 373
———— Tumor under the Tongue 375
———— Aneurism of the Aorta . 430
Cauterization of the Pustules of Small-Pox . . . 68
Cautery, application of the actual, in Erysipelas . . . 441
Comparison of European and Indian Skulls . . . 516
Conformation of the Nervous System in a Cretin . . 66
Contagious nature of Croup, on the 14
Croup, on the various forms of . 9
Cupping Glasses in Poisoned Wounds . . . 67
———— Apparatus, a new . 356

Description of an Instrument for Lithotomy . . . 32
———————— Ophthalmia at Gibraltar and Bombay . 282
———————— of a small Child . 524
Detection of Arsenic in persons poisoned . . . 525
Dhatura, Tincture of, remarks on 522
Diabetes, case of . . . 366
Diminution of Goitre in the Pyrenees . . . 523
Dissection of an Ourang Outang 515

PAGE
Diseases, on the nature and treatment of . . . 53
——— of the Eye, account of 378
——— Lungs . . 431
Dropsy, cases of . . 202
Dyspnœa cured by a Bandage . 173

Effects of Iodine, on the . . 69
——— Strychnia in Epilepsy 173
Efficacy of Lunar Caustic in Inflammation . . . 364
Enlargement of the Mammæ, case of . . . . 260
Ergot of Rye, on the . . 30
Erysipelas, observations on . 288
——— application of the Actual Cautery in the . . 441
Epileptic Convulsions, case of . 103
Exhalations, on the presence of Organic, in the atmosphere . 442
Experience of Dr. Baillie in Fevers 37
Experiments on the secretion of Bile . . . . 354
Extirpation of the Alveolar Arches 355
Eye, on the extirpation of the . 266
——— diseases of, account of . 378

Face, Melanoid Tumor of the . 518
Fallopian Tube, Impregnation in the . . . . 523
Fecula of Plants, on the . . 528
Fever, on the Pathology of . 447
——— and Rheumatism, remarks on . . . . 192
Fevers, on experience in . 37
Forms of Croup, on the various . 9
Fumigation, remarks on . . 264
Further observations on the Oil of Euphorbia Lathyris . . ib.

Gangrena Senilis . . 178
Goitre, diminution of, in the Pyrenees . . . . 523
Gold, Muriate of, in Syphilis . 69
Gonorrhœa, on . . . 266

Headache, severe case of . . 17
———, cured by Purgatives . . . . 463
Hæmorrhage, case of Lithotomy attended with . . . 3
———, Uterine, case of . 458
Heart, unusually small . . 67
Hydrophobia, remarks on . 260
——— in a Horse . . 432

Impregnation in the Fallopian Tube 523
Infants, new-born, marks of Vitality in . . . . 527
Instrument for Lithotomy, description of . . . . 32

PAGE

Iodine, on the effects of . . 69

Large dose of Calomel given to a Child . . . . 433
Lead Plaster, necessity of water in the preparation of . . 267
Lime or Lemon Juice, on the preservation of . . . 442
Lithotomy, case of, attended with Hæmorrhage . . . 3
———, Instrument for the operation of . . . 32
Lithontriptic, Bicarbonate of Soda as a . . . . 522
Lungs, disease of . . . 431

Magnetism, Animal . . 429
Mammæ, case of Enlargement of . 260
Marvellous effects of Acupuncturation . . . . 174
Medical Police . . . 443
Melanoid Tumor of the Face . 518
Metallic Mercury in the Bones . 432
Morbid Tumors on . . 95
Muriate of Gold in Syphilis . 69

Necrosis, remarks on . . 261
Nervous System, on the conformation of, in a Cretin . . 66
Neuralgia of the Sciatic Nerve, remarks on . . . 263
———, case of . . 373
——— from a Wound . . 431
Nose, re-union of a . . 177

Observations on the Small-Pox . 117
——— Pathology . 183
——— the Oil of Euphorbia Lathyris . . . 264
——— Erysipelas . 288
——— on Salivary Calculi . . . . 519
Œsophagus, Abscess of the . 518
Operation for Phymosis . . 523
Ophthalmia, description of, at Gibraltar and Bombay . 282
Opium, analysis of . . 179
Ourang Outang, dissection of . 515
Ovarium, Tumor of the right . 67

Pathology, observations on 183, 447
Phymosis, operation for the . 523
Plants, on the Fecula of . . 528
Poisoned Wounds, Cupping-glasses in . . . . . 67
Poisoning by Opium, case of . 109
Police, Medical . . . 443
Popliteal Aneurism, case of 271, 359
Pregnancy, Abdominal, case of . 178
Preservation of Lime or Lemon Juice . . . . 442

PAGE

Pulmonary Inflammation, on Bleeding on . . . 433
Pustules of Small-Pox, on the Cauterisation of . . 68

Remarks on Fever and Rheumatism . . . . 192
——— Hydrophobia . 260
——— Necrosis . . 261
——— Neuralgia of the Sciatic Nerve . . . 263
——— Fumigation . 264
——— on Secale Cornutum 299, 469
——— the Tincture of Dhatura . . . . 522
Re-union of a Nose . . 177
Rheumatism, acute, Arsenic and Quina in . . . 106
Rhubarb, on the analysis of . 266
Rye, on the Ergot of . . 30

Salivary Calculi, observations on 519
Schirrous affection of the Brain, on 369
Sciatic Nerve, on the Neuralgia of 263
Scurvy in Russia, account of . 465
Secale Cornutum, remarks on, 299, 469
Secretion of Bile, experiments on the . . . . 354
Severe Headache, cured by Purgatives . . . 463

PAGE

Skulls, European and Indian, comparison of . . . 516
Small Child, description of . 524
Small-Pox, case of, after Inoculation . . . . 28
———, observations on . 117
Statistical Medicine 175, 435, 522
Stomach, on the Softening of . 172
Strychnia in Epilepsy, effects of . 173
Syphilis, Muriate of Gold in . 69

Tic Douloureux, cases of . . 123
Tumor of the right Ovarium . 67
——— under the Tongue, case of 375
——— of the Face, Melanoid . 518
Tumors, on morbid . . 95

Urethra, Calculus in the . . 70
Uterus wanting . . . 429
Uterine Hæmorrhage, case of . 458
Utility of Blood-letting, on the . 517

Vitality, marks of, in new-born Infants . . . 527

Water, necessity of, in the preparation of Lead Plaster . . 267
Wound, Neuralgia from a . 431
Wounds, poisoned, Cupping-glasses in . . . . 67

### COLLECTANEA MEDICA.

PAGE

Of Dr. BAILLIE's Experience in Fevers. (From his "Lectures and Observations in Medicine") . . . . . . . . 37

Note on the Actions of the Muscular System. By J. GODMAN, M.D. . 125

On Periodicity in the Actions of the Animal Economy during Health and Disease. By JOHN BELL, M.D. . . . . . . 132

Extracts from a Treatise on Diseases of the Eye, including the Doctrines and Practice of the most eminent modern Surgeons, especially BEER. By G. FRICK, M.D. . . . . . . . 208

On the Mixture and Adulteration of Wine. (From Dr. HENDERSON's "Treatise on Wines") . . . . . . . 304

Report made by M. MAGENDIE to the Academy of Sciences, on the Subject of a Boy Deaf and Dumb from his Birth . . . . . 386

Case of a Wounded Nerve followed by severe Consequences, and cured by removing the Wounded Portion. By G. BELL, Esq. . . . 391

On the Expectoration in Phthisis. By M. ANDRAL . . . 396

An Account of the present State of Medicine in Italy. By W. OPPENHEIM, M.D. . . . . . . . . . . 471

### CRITICAL ANALYSIS.

### DOMESTIC.

On the Action of Calomel: being a Critical Analysis of Part III. of Sketches of the most prevalent Diseases of India, comprising a Treatise on the Epidemic Cholera of the East, &c. By JAMES ANNESLEY, Esq. &c. . 40

Researches into the Nature and Treatment of Dropsy in the Brain, Chest, Abdomen, Ovarium, and Skin; in which a more correct and consistent Pathology of these Diseases is attempted to be Established, and a New and more successful Method of Treating them, recommended and explained. By JOSEPH AYRE, M.D. . . . . . . 138

An Essay on the Application of Lunar Caustic in the Cure of certain Wounds and Ulcers. By JOHN HIGGINBOTTOM, Nottingham . . . . 154

A Practical Treatise on Diabetes; with Observations on the Tabes Diuretica, or Urinary Consumption, especially as it occurs in Children, and on Urinary Fluxes in general. With an Appendix of Dissections and Cases, and a Postscript of practical Directions for examining the Urine in those Diseases. By ROBERT VENABLES, M.D. . . . . . 222

An Introduction to the Use of the Stethoscope; with its Application to the Diagnosis in Diseases of the Thoracic Viscera. By WM. STOKES, M.D. 314, 418

Transactions of the Medical and Physical Society of Calcutta. Vol. I. . 401

Practical Observations on the Convulsions of Infants. By JOHN NORTH, Surgeon Accoucheur . . . . . . . . . 486

### FOREIGN.

Memoires sur la Nature et le Traitement de plusieurs Maladies. Par M. PORTAL, &c. &c. . . . . . . . . 53

A Treatise on the Physical and Medical Treatment of Children. By WM. P. DEWEES, M.D. Member of the American Philosophical Society of Philadelphia, &c. . . . . . . . . . 161

Elements of Physiology. By K. A. RUDOLPHI, M.D. . . . . 231

PAGE

Recherches Anatomico-Pathologiques sur la Phthisie. Par P. C. A. Louis, M.D. . . . . . . . . . 247, 339

New Method of curing Trichiasis: a Memoir, by Professor A. Vacca Berlinghieri . . . . . . . . 333

An Elementary Compendium of Physiology, for the Use of Students. By F. Magendie, M.D. . . . . . . . . 425

An Elementary Treatise on Diagnosis, Prognosis, and Therapeutical Indications; or a Course of Clinical Medicine. By M. L. Rostan, M.D. . 503

### MISCELLANEOUS INTELLIGENCE.

Society for the Diffusion of Obstetrical Knowledge . . . 71

Prize Essay . . . . . . . . 180

Medical, Clerical, and General Life Assurance Society . . . 267

Medical Police . . . . . . . . 443

Regulations for the Medical Service of the Navy . . . 444

Breeding of Leeches . . . . . . 528

### MONTHLY CATALOGUE OF BOOKS.

Pages 73, 181, 269, 357, 445, 529.

### METEOROLOGICAL TABLES.

Pages 94, 182, 270, 358, 446, 530.

### NOTICES TO CORRESPONDENTS.

Pages 94, 182, 270, 358, 446, 530.

### ERRATA.

Pages 94, 358.

### PLATES.

Mr. Shaw's Case of Lithotomy, attended with Hemorrhage . . 1
Mr. Chevalier's General Observations on Morbid Tumors . . 95

### NAMES OF AUTHORS,

*Of whose Works, Observations, &c. either a detailed Account, or more or less general View, is given in*

### THE FIFTY-FIFTH VOLUME

### OF THE LONDON MEDICAL AND PHYSICAL JOURNAL.

PAGE

ADAM, Dr. on the exhibition of Phosphorus . . . . 406

——— on a Tumor in a native of India . . . . 413

——— on the Tincture of Dhatura . . . . . 522

ANDRAL, M. on Expectoration in Phthisis . . . . 396

ANNESLEY, Dr. on the use of Calomel . . . . . 40

ARNOTT, Mr. case of Popliteal Aneurism . . . . . 271

ASHBURY, Mr. VALE, case of Nervous Excitement . . . 16

AYRE, Dr. on Dropsy . . . . . . . 138

BACOT, Mr. case of Tumor . . . . . . 375

BAGNOLD, Captain, on the Preservation of Lemon Juice . . 442

BAKER, Mr. on the Nux Vomica . . . . . 412

——— on a singularly small Child . . . . . 524

BAILLIE, Dr. his Experience in Fevers . . . . . 37

BALLY, M. on the Oil of Euphorbia Lathyris . . . . 264

BARRY, Dr. on Cupping-glasses in Poisoned Wounds . . . 69

BELL, Dr. on Periodicity . . . . . . . 132

BELL, Mr. G. case of Wounded Nerve . . . . . 391

BENNABEN, Dr. on Gold in Syphilis . . . . . . 69

BENZA, Dr. case of Popliteal Aneurism . . . . 359

BERNT, Dr. on the Marks of Vitality in New-born Infants . . 527

BERZELIUS, M. method of Detecting Arsenic . . . . 525

BIZIO, M. on the Atmosphere . . . . . . 442

BLACKETT, Mr. on Tic Doloureux . . . . . 122

——— on Erysipelas . . . . . . 289

BLAKE, Dr. on an improved Instrument for Lithotomy . . . 32

——— case of Convulsions . . . . . . 163

BOULU, M. on Calculus in the Urethra . . . . . 70

BOYLE, Mr. case of Anchylosis of the Knee . . . . 112

BRETON, Mr. on a Wound in the Stomach . . . . 405

BROFFIERRES, Dr. on the Effects of Strychnia . . . . 173

BROUGHTON, Mr. case of Neuralgia . . . . . 373

BROWN, Mr. on Fever and Rheumatism . . . . . 192

BURNARD, Mr. case of Fungoid Disease . . . . 411

BUTH, Mr. case of Abdominal Pregnancy . . . . 170

CARBARO, Dr. on Acupuncturation . . . . . . 174

CHATELAIN, M. on the Breeding of Leeches . . . . 528

CHEVALIER, Mr. on Morbid Tumors . . . . . . 95

CLARK, Mr. his new Cupping Apparatus . . . . 356

——— on the Ergot of Rye . . . . . 30

CULLERIER, Mr. on Gonorrhœa . . . . . . . 266

DAVY, Dr. cases of Dropsy . . . . . . 202

PAGE

Delfix, M. on Enlargement of the Mammæ . . . . 260
Dewees, Dr. on the Treatment of Children . . . . 161
Dupuytren, M. on Gangrena Senilis . . . . . 178

Elliotson, Dr. on the Use of Carbonate of Iron . . . 72

Frick, Dr. on Diseases of the Iris . . - . . 208
Frost, Mr. on Erysipelas from the Use of the Rhus Toxicodendron . 116

Gilby, Dr. on the Effects of a Bandage . . . . 173
Gobert, Dr. on Blood-letting . . . . . . 517
Godman, Dr. on the Muscular System . . . . . 125
Granville, Dr. case of severe and protracted Headache . . 17
Green, Mr. on Fumigation . . . . . . 265
Gregory, Dr. on Small-Pox . . . . . . 117

Henderson, Dr. on the Adulteration of Wines . . . . 304
Higginbottom, Mr. on the Application of the Lunar Caustic . 155, 364

Jewel, Mr. case of Poisoning by Opium . . . . 109

Laennec, M. on Disease of the Lungs . . . . . 431

Larrey, M. on the Cautery in Erysipelas . . . . 441

Lefevre, Dr. case of Diabetes . . . . . . 366

Lee, Dr. account of Scurvy . . . . . . 465

Lisfranc, M. on Neuralgia from a Wound . . . . 431

———— on Extirpation of the Uterus . . . . 442

Locher-Balber, M. on the Effects of Iodine . . . . 69

Louis, Dr. on Phthisis . . . . . . . 247, 339

Mackenzie, Professor, on the Secale Cornutum . . . 469

Macleod, Dr. case of Hydrophobia in a Horse . . . 432

———— on large Doses of Calomel . . . . 433

Magendie, M. Report on a Deaf and Dumb Boy . . . 386

———— Treatise on Physiology . . . . . 425

Masseau, M. case of unusually small Heart . . . . 67

Maynard, Mr. on Aneurism of the Aorta . . . . 430

Nani, M. on the Analysis of Rhubarb . . . . . 266
Naudin, M. on Hydrophobia . . . . . . 260
North, Mr. on the Convulsions of Children . . . . 486

Oppenheim, Dr. on the State of Medicine in Italy . . . 471

Oswald, Mr. case of Abdominal Dropsy . . . . 275

Patterson, Dr. on the Comparison of Human Skulls . . . 516

Playfair, Mr. on the Madár . . . . . . 409

Portal, M. on the Treatment of Disease . . . . 53

Pretty, Mr. on the various Forms of Croup . . . . 9

Ramisch, Dr. on Softening of the Stomach . . . . 172
Raspail, M. on the Fecula of Plants . . . . . 528
Regnoli, Dr. on Extirpation of the Jaw . . . . 355
Renaudin, M. case of Uterus wanting . . . . 429
Renauldin, M. on Plague and Yellow Fever . . . . 522
Reveille-Parise, M. on Sciatica . . . . . 263
Richards, Mr. case of severe Headache . . . . 463
———— case of Small-Pox after Small-Pox Inoculation . . 28
Richmond, Mr. on Ophthalmia . . . . . . 283, 378
Richter, Professor, on Necrosis . . . . . 261
Ricord, M. case of Melanoid Tumor of the Face . . . 518
Rostan, M. on Prognosis, Diagnosis, &c. . . . . 503

| | PAGE |
|---|---|
| ROBIQUET, M. on the Analysis of Opium . . . . | 179 |
| ———— on the Bicarbonate of Soda . . . . | 522 |
| ROULLIN, M. on Goitre in the Pyrenees . . . . . | 523 |
| RUDOLPHI, Dr. on Physiology . . . . . . | 231 |
| SCHIFFNER, Dr. on the Nervous System in a Cretin . . . | 66 |
| SEGALAS, M. Experiments on Poisoning . . . . | 516 |
| SERUS and BRETONNEAU, MM. on Cauterization in Small-Pox . . | 69 |
| SHAW, Mr. case of Lithotomy . . . . . . . | 3 |
| STOKER, Dr. observations in Pathology . . . . | 183, 447 |
| STOKES, Dr. on the Stethoscope . . . . . | 314, 418 |
| SYM, Mr. on the Contagious Nature of Croup . . . . | 14 |
| TWINING, Mr. on the Worm in the Eye of the Horse . . . | 415 |
| VACCA, Professor, on Trichiasis . . . . . . . | 333 |
| VENABLES, Dr. on Diabetes . . . . . . | 222 |
| VETTU, M. case of Tumor of the Ovarium . . . . | 67 |
| WADE, Mr. case of Apoplexy . . . . . . | 190 |
| ———— on Schirrous Affection of the Brain . . . | 369 |
| WALLER, Mr. on Secale Cornutum . . . . . | 299 |
| ———— case of Uterine Hemorrhage . . . . | 458 |
| WHITING, Dr. on Arsenic in Acute Rheumatism . . . . | 106 |
| WILSON, Mr. on Lepra as known to the Hindus . . . . | 404 |

